# The impact of epilepsy and antiseizure medications on pregnancy and neonatal outcomes: A nationwide cohort study

**DOI:** 10.1002/brb3.3287

**Published:** 2023-10-14

**Authors:** Cheng‐Yen Kuo, Chang‐Fu Kuo, Lai‐Chu See, Meng‐Jiung Chiou, Po‐Cheng Hung, Jainn‐Jim Lin, Kuang‐Lin Lin, Huei‐Shyong Wang, I‐Jun Chou

**Affiliations:** ^1^ Division of Pediatric Neurology Chang Gung Memorial Hospital Taoyuan Taiwan; ^2^ Division of Rheumatology, Allergy and Immunology Chang Gung Memorial Hospital Taoyuan Taiwan; ^3^ Division of Rheumatology, Orthopaedics, and Dermatology, School of Medicine University of Nottingham Nottingham UK; ^4^ Center for Artificial Intelligence in Medicine Chang Gung Memorial Hospital Taoyuan Taiwan; ^5^ Department of Public Health College of Medicine, Biostatistics Core Laboratory, Molecular Medicine Research Centre Chang Gung University Taoyuan Taiwan

**Keywords:** antiseizure medications (ASMs), epilepsy, outcome, pregnancy, women with epilepsy

## Abstract

**Purpose:**

Our objective was to assess the adverse outcomes during pregnancy, as well as for the fetus and neonates, in women with epilepsy, both with and without the use of antiseizure medications (ASMs).

**Methods:**

A cohort of singleton pregnancies between January 1, 2004 and December 31, 2014 was identified using the Taiwan National Health Database. The pregnancies were categorized into ASM exposure, ASM nonexposure, and control (consisting of women without an epilepsy diagnosis) groups. We recorded adverse outcomes in neonates and documented pregnancy complications. The generalized estimating equation with logit link was used to estimate adjusted odds ratios.

**Results:**

There were 629 singleton pregnancies in the group exposed to ASMs, 771 in the epilepsy group without ASM exposure, and 2,004,479 in the control group. Women with epilepsy had a significantly higher risk of puerperal cerebrovascular diseases (adjusted odds ratios in the exposure and nonexposure groups = 54.46 and 20.37, respectively), respiratory distress syndrome (5.1 and 2.99), mortality (3.15 and 3.22), sepsis (2.67 and 2.54), pregnancy‐related hypertension (1.71 and 1.8), preeclampsia (1.87 and 1.79), cesarean delivery (1.72 and 2.15), and preterm labor (1.38 and 1.56). The use of ASMs may increase the risk of eclampsia (adjusted odds ratio = 12.27). Compared to controls, fetuses/neonates born to women with epilepsy had a higher risk of unexplained stillbirth (adjusted odds ratios in the exposure and nonexposure groups = 2.51 and 2.37, respectively), congenital anomaly (1.37 and 1.33), central nervous system malformation (3.57 and 2.25), low birth weight (1.90 and 1.97), and a low Apgar score at 5 min (2.63 and 1.3). The use of ASMs may introduce an additional risk of small for gestational age; the adjusted odds ratio was 1.51.

**Conclusion:**

Women with epilepsy, irrespective of their exposure to ASMs, had a slightly elevated risk of pregnancy and perinatal complications. Puerperal cerebrovascular diseases may be a hidden risk for women with epilepsy.

## Graphical abstract

Comparison of pregnancy outcomes with or without use antiepileptic drugs in pregnant women with epilepsy.

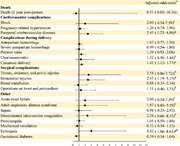

## 1 INTRODUCTION

Epilepsy is a common condition with a lifetime prevalence of 6.38 cases per 1000 individuals (Fiest et al., 2017). Approximately one‐fifth of women with epilepsy are of childbearing age (Wallace et al., 1998). Recent studies have revealed that the pregnancy risks for women with epilepsy are not solely linked to seizures during pregnancy and antiseizure medications (ASMs) but are also associated with epilepsy itself. There is a 5‐ to 10‐fold increased risk of mortality during childbirth (Kapoor & Wallace, 2014; Macdonald et al., 2015; Yucel et al., 2021), as well as of pregnancy‐related complications such as anemia, gestational hypertension, preeclampsia, premature rupture of membranes, premature delivery, cesarean section, postpartum hemorrhage (Huang et al., 2020; Razaz et al., 2017; Viale et al., 2015), infection, stillbirth, and small for gestational age at birth (Razaz et al., 2017). Epilepsy, even without the use of ASMs, increases the risk of congenital malformations in newborns and fetuses (Razaz et al., 2017).

Moreover, seizures during pregnancy are associated with an increased risk of low birth weight, preterm delivery, and small gestational age (Chen et al., 2009). The teratogenic effects of specific ASMs, such as valproate, topiramate, and phenobarbital, are well‐established. These medications can lead to conditions like congenital heart disease, hypospadias, neural tube defects, microcephaly, and fetal growth restriction (Tomson et al., 2015; Veiby et al., 2014). Such factors add to the complexity of managing epilepsy during pregnancy. Achieving the best pregnancy outcome requires careful planning, using ASMs with lower teratogenic risks, and achieving improved seizure control before conception (Abe et al., 2014; Zhang et al., 2020).

Many of these complications can be monitored proactively and carefully managed to prevent additional mortality and morbidity in women with epilepsy and their offspring. Therefore, we conducted a nationwide cohort study in Taiwan to determine the incidence of pregnancy and postpartum complications, as well as adverse fetal‐neonatal outcomes, among women with singleton pregnancies. We compared these outcomes between women with epilepsy and a control group without epilepsy. Our analysis investigated whether these complications were linked to epilepsy, ASMs, or both.

## 2 METHODS

### 2.1 Study design and cohort

We conducted a nationwide cohort study using data prospectively collected from the Taiwan National Health Insurance Research Database (NHIRD), the Taiwanese Birth Registry, and the Taiwanese Death Registry. The study population included singleton pregnancies with live births or stillbirths between January 1, 2004 and December 31, 2014. We excluded individuals with multiple births, maternal age < 18 or > 45 years, intervals between two deliveries of < 6 months or > 20 years, and pregnancies with a gestational age of ≤ 20 weeks. We also excluded patients diagnosed with epilepsy who had no recorded prescriptions for ASMs, as well as those prescribed ASMs but without a diagnostic code for epilepsy (Figure S1). The study cohort was divided into epilepsy with ASM (exposure), epilepsy without ASM (nonexposure), and normal control groups.

To assess the teratogenic effect of ASMs, the exposure group comprised women with epilepsy prescribed ASMs within 3 months before or after their last menstrual period, which corresponds to the critical periods for major organ formation. Those diagnosed with epilepsy but not prescribed ASMs in this timeframe constituted the nonexposure group. The control group included women without an epilepsy diagnosis before pregnancy (Figure S2).

We collected data on epilepsy diagnoses, ASM usage, maternal comorbidities, pregnancy and delivery outcomes, fetal‐neonatal outcomes, maternal mortality, and delivery and postpartum outcomes up to 1 year postpartum. After adjusting for covariates, we compared the outcomes among the three groups.

### 2.2 Antiseizure medications of interest

We analyzed 15 ASMs: acetazolamide, carbamazepine, clobazam, clonazepam, gabapentin, lamotrigine, levetiracetam, oxcarbazepine, phenobarbital, phenytoin, pregabalin, topiramate, valproate, vigabatrin, and zonisamide. The anatomical therapeutic chemical (ATC) codes of these medications are listed in Table S1.

### 2.3 Epilepsy and outcomes of interest based on International Classification of Disease (ICD) codes

Epilepsy was considered present when the two following conditions were met: at least three outpatient visits with a clinical diagnosis of epilepsy (ICD‐9 code 345) or at least one hospitalization with a principal diagnosis of epilepsy, and receiving at least one prescription for ASMs at clinical visits (Chou et al., 2021).

We assessed a broad range of pregnancy and fetal‐neonatal outcomes based on physician diagnoses, neonatal delivery records, and maternal discharge notes from delivery‐related hospital stays. Outcomes were categorized using ICD‐9 (Table S2). The accuracy of the coding has been validated (Lu et al., 2001; Lu et al., 2000).

Pregnancy outcomes were classified as death (≤ 30 days or ≤ 1 year postpartum), cardiovascular complications, preeclampsia, eclampsia, complications during delivery, surgical complications, or other (acute renal failure, adult respiratory distress syndrome, pulmonary edema, sepsis, disseminated intravascular coagulation, mechanical ventilation, or gestational diabetes). Maternal deaths were ascertained using the National Death Registry (NDR) in Taiwan, which records the causes of death for all deceased citizens.

The fetal‐neonatal outcomes include stillbirth, low birth weight (< 2500 g), prematurity (< 37 weeks), small (or large) birthweight for gestational age (small, < 10th percentile for the same gestational age; appropriate, 10th to 90th percentile for gestational age; large, > 90th percentile for gestational age), Apgar scores at 1 and 5 min after birth, fetal distress, fetal abnormalities, central nervous system (CNS) malformations, chromosomal abnormalities, suspected damage due to viral or other diseases in the mother, suspected damage due to drugs or radiation, decreased fetal movements, and other/unspecified abnormalities. Small and large for gestational age were based on a nomogram of all live births recorded between 2004 and 2014 in the National Birth Registry (NBR) (Figure S3). According to the system described by King‐Hele et al. (2009), stillbirths were categorized as explained or unexplained.

Maternal covariates were maternal age, maternal country of origin, place of residence, income level, occupation, obstetric history, and Charlson comorbidity index (CCI). Infant covariates were birth weight, sex, and gestational age. The place of residence was assigned as one of 369 towns or districts in Taiwan. The levels of urbanization of these 369 towns or districts were classified as urban, suburban, or rural. Occupations were classified into the following five categories: (a) civil servants, teachers, and military personnel/veterans; (b) professionals and nonmanual workers; (c) manual workers; (d) other; and (e) dependents. Income levels were estimated using employee payrolls and employer business incomes. We categorized income levels into sex‐specific income quintiles.

We assessed maternal comorbidity using the CCI during the 3 years before the last menstrual period. The CCI comprises 17 mortality‐related disease categories (myocardial infarction, congestive heart failure, peripheral vascular disease, cerebrovascular disease, dementia, chronic pulmonary disease, rheumatological disease, peptic ulcer disease, mild liver disease, moderate or severe liver disease, diabetes mellitus, diabetes mellitus with chronic complications, renal diseases, any malignancy, metastatic solid tumor, and human immunodeficiency virus infection). We used the validated version of the ICD‐9 developed by Deyo et al. (1992).

### 2.4 Data source

This nationwide cohort study was based on the Taiwan NHIRD, linked to the NBR and NDR. The National Health Insurance Program was established in 1995 and covers 99.5% of the Taiwanese population. Upon request, all information in the database can be internally and externally linked to other databases (e.g., civil registration, death registry, birth registry, and other government‐held data), using a unique personal identifier encrypted before releasing the data to researchers.

In Taiwan, prenatal and postnatal care is reimbursed by the NHI, including clinical diagnosis, physical examinations, laboratory tests, fetal ultrasound in each trimester, health education and guidance, delivery, and infant and postpartum care. The NBR contains records of live births and stillbirths (> 20 weeks old or > 500 g weight) since 2001. Variables in the registry include demographics, pregnancy conditions, newborn conditions, and spousal demographics. The quality of these data has been validated in terms of pregnancy and fetal‐neonatal outcomes. The birth registry accurately reports sex, birth order, gestational age, neonatal abnormalities, Apgar score, and birth weight. High concordance between the NBR and a retrospective chart review was indicated by a kappa statistic of > 85%. The kappa value ranged from 0.92 to 0.96 for birth weight and gestational age as categorical variables (King‐Hele et al., 2009).

### 2.5 Statistical analysis

We compared each outcome among the three groups using a generalized estimating equation with a logit to estimate the adjusted odds ratio and 95% confidence intervals (CIs) because some women had more than one pregnancy during the study period. The within‐subject correlation was based on an autoregressive structure in generalized estimating equation analysis (Zeger & Liang, 1992). Bayesian logistic regression using data augmentation was performed to avoid sparse data bias because of the low rates of some outcomes (< 5) (Sander Greenland, 2007, 2016). The prior defined analysis was adjusted for age, infant sex, CCI, urbanization, income, occupation, birth year, maternal nationality, maternal smoking, and maternal alcohol consumption. A two‐sided test at a 5% level of significance was used for all statistical hypotheses. Statistical analysis was performed using SAS software, version 9.3 (SAS Institute, Cary, NC, USA).

## 3 RESULTS

Of the 2,005,879 singleton pregnancies documented, approximately 1400 (0.07%) involved women with epilepsy preexisting before conception. The nonexposure and exposure groups accounted for 771 (0.04%) and 629 (0.03%) pregnancies, respectively. The average maternal ages at the time of pregnancy for the control, nonexposure, and exposure groups were 30.12, 29.44, and 29.53 years, respectively. In all three groups, urban residency was predominant. A notable proportion of epileptic patients, ranging from 74% to 76%, fell within income level quintiles 1−3, surpassing the proportion in the control group (60%). The majority of women across the groups (84.9%−96.8%) had no comorbidities (CCI = 0). Notably, both the exposure and nonexposure groups had higher proportions of individuals with a CCI ≥ 1 (15.1% and 9.34%, respectively) than the control group (3.18%) (Table 1). Significant disparities were observed among the groups in age distribution, residential location, income tier, occupation, and comorbidity rate (*p* < .001). Consequently, these variables were adjusted for in the subsequent analysis of pregnancy and fetal‐neonatal outcomes (Table 1). In the group exposed to ASMs, most women were on monotherapy (63.8%), 23.7% were taking two ASMs, and 12.5% were taking three ASMs. The three most frequently prescribed ASMS were carbamazepine (37.7%), lamotrigine (23.7%), and valproate (20.9%) (Table S3). The mean doses of carbamazepine and valproate were 600 and 794 mg/day, respectively.

**TABLE 1:** Baseline characteristics of pregnant women without epilepsy (control), with epilepsy and taking (exposure) or not taking (nonexposure) antiseizure medications.

	Control (*n* = 2,004,479)		Nonexposure (*n* = 771)		Exposure (*n* = 629)		*p* Value
Age at pregnancy, mean (SD), years	30.12	(4.74)	29.44	(4.98)	29.53	(4.87)	<.0001
<25	281,774	(14.06)	160	(20.75)	115	(18.28)	
25–34	1,402,000	(69.94)	502	(65.11)	422	(67.09)	
>34	320,705	(16.00)	109	(14.14)	92	(14.63)	
Male infant	1,044,287	(52.10)	407	(52.79)	320	(50.87)	.7693
Place of residence, No. (%)							<.0001
Urban	1,260,286	(62.87)	449	(58.24)	360	(57.23)	
Suburban	604,830	(30.17)	234	(30.35)	207	(32.91)	
Rural	127,215	(6.35)	86	(11.15)	60	(9.54)	
Unknown	12,148	(0.61)	2	(0.26)	2	(0.32)	
Income levels, No. (%)							<.0001
Quintile 1	406,008	(20.26)	215	(27.89)	179	(28.46)	
Quintile 2	451,598	(22.53)	168	(21.79)	173	(27.50)	
Quintile 3	345,835	(17.25)	187	(24.25)	123	(19.55)	
Quintile 4	411,097	(20.51)	104	(13.49)	91	(14.47)	
Quintile 5	389,772	(19.45)	97	(12.58)	61	(9.70)	
Unknown	169	(0.01)	0	(0.00)	2	(0.32)	
Occupation, No. (%)							<.0001
Dependents	528,551	(26.37)	207	(26.85)	196	(31.16)	
Civil servants, teachers, military personnel, and veterans	101,293	(5.05)	24	(3.11)	16	(2.54)	
Nonmanual workers and professionals	796,878	(39.75)	215	(27.89)	166	(26.39)	
Manual workers	333,741	(16.65)	188	(24.38)	153	(24.32)	
Other	244,016	(12.17)	137	(17.77)	98	(15.58)	
Charlson comorbidity index							<.0001
0	1,940,676	(96.82)	699	(90.66)	534	(84.90)	
1	55,340	(2.76)	54	(7.00)	70	(11.13)	
≥2	8463	(0.42)	18	(2.33)	25	(3.97)	

### 3.1 Adverse pregnancy outcomes

The three most common adverse pregnancy outcomes were cesarean delivery, antepartum hemorrhage, and preterm labor. Less common complications were puerperal cerebrovascular diseases, sepsis, and adult respiratory distress syndrome. The following complications had small event numbers (≤ 5) despite being significantly more frequent in women with epilepsy (regardless of ASM exposure) than in the controls: postpartum maternal deaths (three women), arrhythmias requiring cardioversion (one woman), severe anesthesia complications (two women), thorax abdomen and pelvis injuries (five women), operations on heart and pericardium (five women), acute renal failure (two women), and disseminated intravascular coagulation (four women).

Compared with the controls, women with epilepsy had markedly odds ratios for several negative health outcomes. Specifically, the adjusted odds ratios for puerperal cerebrovascular diseases in the exposure and nonexposure cohorts were 54.46 (95% CI: 34.20−86.74) and 20.37 (95% CI: 11.90−24.87), respectively. For adult respiratory distress syndrome, the ratios were 5.1 (95% CI: 2.08−12.49) and 2.99 (95% CI: 1.16−7.75), respectively. The odds ratios for sepsis were 2.67 (95% CI: 1.27−5.62) and 2.54 (95% CI: 1.18−5.51), those for preeclampsia were 1.79 (95% CI: 1.15−2.80) and 1.87 (95% CI: 1.27−2.77), those for pregnancy‐induced hypertension were 1.80 (95% CI: 1.24−2.62) and 1.71 (95% CI: 1.23−2.39), those for cesarean delivery were 2.15 (95% CI: 1.82−2.54) and 1.72 (95% CI: 1.46−2.01), and those for preterm labor were 1.84 (95% CI: 1.37−2.47) and 1.42 (95% CI: 1.06−1.90), respectively (Table 2).

**TABLE 2:** Pregnancy adverse outcomes among women without epilepsy (control), with epilepsy and taking (exposure) or not taking (nonexposure) antiseizure medications.

	No. of events (%)						Crude odds ratio (Control as reference group)				Adjusted odds ratio^a^ (Control as reference group)			
	Control (*n* = 2,004,479)		Nonexposure (*n* = 771)		Exposure (*n* = 629)		Nonexposure		Exposure		Nonexposure		Exposure	
**Death**														
Death ≤30 days postpartum	262	(0.01)	0	(0.00)	1	(0.16)	NA		12.18	(1.71–86.99)*	NA		9.83	(1.39–69.71)*
							NA		2.82	(0.80–9.96) ^b^			2.75	(0.79–9.53) ^b^
Death ≤1 year postpartum	620	(0.03)	1	(0.13)	1	(0.16)	8.44	(2.10–33.84)	10.33	(2.57–41.46)*	6.51	(1.59–26.67)*	6.97	(1.71–28.45)*
							3.42	(1.09–10.80)* ^b^	3.62	(1.11–11.74)* ^b^	3.15	(1.04–9.53)* ^b^	3.22	(1.05–9.85)* ^b^
**Cardiovascular complications**														
Acute myocardial	92	(0.00)	0	(0.00)	0	(0.00)	NA		NA		NA		NA	
Shock	1196	(0.06)	1	(0.13)	1	(0.16)	1.85	(0.21–16.19)	2.56	(0.36–17.98)	1.54	(0.18–13.20)	2.10	(0.29–14.96)
							2.06	(0.69–6.17) ^b^	2.18	(0.71–6.68) ^b^	1.95	(0.66–5.72) ^b^	2.04	(0.68–6.08) ^b^
Arrhythmias requiring cardioversion	672	(0.03)	1	(0.13)	0	(0.00)	5.76	(1.14–29.11)*	NA		4.94	(1.05–23.32)*	NA	
							3.35	(1.07–10.44)* ^b^	NA		3.1	(1.03–9.31)* ^b^	NA	
Pregnancy‐related hypertension	51,388	(2.56)	38	(4.93)	36	(5.72)	2.03	(1.46–2.83)*	2.20	(1.54–3.14)*	1.71	(1.23–2.39)*	1.80	(1.24–2.62)*
Preeclampsia	34,462	(1.72)	28	(3.63)	24	(3.82)	2.19	(1.48–3.23)*	2.20	(1.44–3.37)*	1.87	(1.27–2.77)*	1.79	(1.15–2.80)*
Puerperal cerebrovascular diseases	1405	(0.07)	14	(1.82)	31	(4.93)	25.40	(14.93–43.22)*	71.93	(46.63–110.96)*	20.37	(11.90–34.87)*	54.46	(34.20–86.74)*
Eclampsia	1414	(0.07)	1	(0.13)	7	(1.11)	3.52	(0.78–15.78)	14.77	(6.10–35.77)*	3.05	(0.65–14.32)	12.27	(4.95–30.42)*
Thromboembolism	206	(0.01)	1	(0.13)	0	(0.00)	12.91	(1.81–92.04)*	NA		9.93	(1.37–71.84)*	NA	
							2.83	(0.80–10.01) ^b^	NA		2.76	(0.79–9.57) ^b^	NA	
**Complications during delivery**														
Amniotic fluid embolism	218	(0.01)	0	(0.00)	0	(0.00)	NA		NA		NA		NA	
Antepartum hemorrhage	178,033	(8.88)	87	(11.28)	76	(12.08)	1.30	(1.03–1.64)*	1.39	(1.08–1.80)*	1.24	(0.98–1.57)	1.34	(1.04–1.73)*
Severe postpartum hemorrhage	54,990	(2.74)	28	(3.63)	20	(3.18)	1.37	(0.94–1.99)	1.17	(0.74–1.84)	1.18	(0.81–1.71)	1.02	(0.65–1.61)
Preterm labor	93,776	(4.68)	54	(7.00)	58	(9.22)	1.53	(1.14–2.05)*	2.02	(1.51–2.71)*	1.42	(1.06–1.90)*	1.84	(1.37–2.47)*
Premature rupture of membranes	76	(0.00)	0	(0.00)	0	(0.00)	NA		NA		NA		NA	
Chorioamnionitis	7519	(0.38)	6	(0.78)	3	(0.48)	2.14	(0.96–4.75)	1.32	(0.43–4.06)	1.94	(0.87–4.31)	1.22	(0.39–3.74)
							2.06	(1.04–4.10)* ^b^	1.53	(0.68–3.46) ^b^	1.92	(0.97–3.79) ^b^	1.45	(0.65–3.25) ^b^
Cesarean delivery	667,984	(33.32)	353	(45.78)	347	(55.17)	1.76	(1.51–2.05)*	2.17	(1.85–2.55)*	1.72	(1.46–2.01)*	2.15	(1.82–2.54)*
Induction of labor	84	(0.00)	0	(0.00)	0	(0.00)	NA		NA		NA		NA	
**Surgical complications**														
Severe anesthesia complications	215	(0.01)	0	(0.00)	2	(0.32)	NA		14.85	(2.09–105.45)*	NA		12.44	(1.68–92.24)*
							NA		2.87	(0.80–10.25) ^b^	NA		2.83	(0.80–10.00) ^b^
Thorax, abdomen, and pelvis injuries	819	(0.04)	1	(0.13)	4	(0.64)	3.32	(0.48–23.08)	15.81	(5.92–42.26)*	2.61	(0.37–18.37)	12.30	(4.55–33.25)*
							2.27	(0.73–7.09) ^b^	6.39	(2.16–18.90)* ^b^	2.13	(0.70–6.48) ^b^	5.56	(1.98–15.60)* ^b^
Intracranial injuries	4363	(0.22)	12	(1.56)	25	(3.97)	7.17	(3.85–13.35)*	18.79	(12.40–28.47)*	5.56	(2.97–10.40)*	13.95	(9.20–21.14)*
Blood transfusion	12,462	(0.62)	10	(1.30)	8	(1.27)	1.95	(1.02–3.74)*	2.07	(1.02–4.17)*	1.64	(0.85–3.15)	1.74	(0.85–3.54)
Hysterectomy	1589	(0.08)	0	(0.00)	1	(0.16)	NA		1.94	(0.27–13.72)	NA		1.83	(0.25–13.16)
							NA		2	(0.67–5.94) ^b^	NA		1.92	(0.66–5.63) ^b^
Operations on heart and pericardium	1983	(0.10)	4	(0.52)	1	(0.16)	4.99	(1.78–14.00)*	3.32	(0.84–13.18)	3.35	(1.16–9.71)*	2.23	(0.54–9.21)
							3.58	(1.46–8.77)* ^b^	2.46	(0.90–6.78) ^b^	2.81	(1.21–6.54)* ^b^	2.05	(0.79–5.36) ^b^
**Other**														
Acute renal failure	483	(0.02)	1	(0.13)	1	(0.16)	5.68	(0.82–39.23)	13.66	(3.43–54.45)*	4.05	(0.66–24.65)	7.89	(1.89–33.05)*
							2.54	(0.77–8.38) ^b^	3.84	(1.14–12.92)* ^b^	2.31	(0.73–7.29) ^b^	3.2	(1.04–9.80)* ^b^
Adult respiratory distress syndrome	1208	(0.06)	3	(0.39)	5	(0.79)	6.24	(2.02–19.29)*	13.17	(5.38–32.23)*	4.17	(1.32–13.12)*	8.85	(3.62–21.64)*
							3.64	(1.32–10.01)* ^b^	6.75	(2.57–17.71)* ^b^	2.99	(1.16–7.75)* ^b^	5.1	(2.08–12.49)* ^b^
Sepsis	6547	(0.33)	8	(1.04)	7	(1.11)	3.09	(1.43–6.71)*	3.34	(1.58–7.05)*	2.54	(1.18–5.51)*	2.67	(1.27–5.62)*
Disseminated intravascular coagulation	1556	(0.08)	3	(0.39)	1	(0.16)	4.34	(1.26–15.00)*	4.04	(1.02–16.00)*	3.98	(1.22–13.02)*	3.52	(0.87–14.17)
							3.24	(1.22–8.57)* ^b^	2.71	(0.95–7.70) ^b^	3.11	(1.19–8.13) ^b^	2.56	(0.92–7.13) ^b^
Pulmonary edema	309	(0.02)	0	(0.00)	0	(0.00)	NA		NA		NA		NA	
Mechanical ventilation	24,537	(1.22)	19	(2.46)	14	(2.23)	1.95	(1.20–3.19)*	1.84	(1.08–3.12)*	1.58	(0.97–2.59)	1.53	(0.90–2.61)
							2.6	(0.93–7.28) ^b^	9.27	(3.98–21.58)* ^b^	2.44	(0.89–6.69) ^b^	7.73	(3.41–17.51)* ^b^
Gestational diabetes	126,229	(6.30)	39	(5.06)	21	(3.34)	0.82	(0.59–1.13)	0.52	(0.34–0.81)*	0.80	(0.57–1.11)	0.52	(0.34–0.81)*

^a^ Adjusted for age, infant sex, Charlson comorbidity index, urbanization, income, occupation, birth year, maternal nationality, maternal smoking, maternal alcohol consumption.

^b^ Bayesian logistic regression using data augmentation to avoid sparse data bias because of the low occurrence of outcomes (number < 5).

*p < .05.

Abbreviation: N/A, not applicable.

Women diagnosed with epilepsy and exposed to ASMs had a higher risk of eclampsia, with an adjusted odds ratio of 12.27 (95% CI: 4.95−30.42) compared to the controls. Epileptic women without ASM exposure did not have a comparable risk, with an odds ratio of 3.05 (95% CI: 0.65−14.32).

**FIGURE 1:**
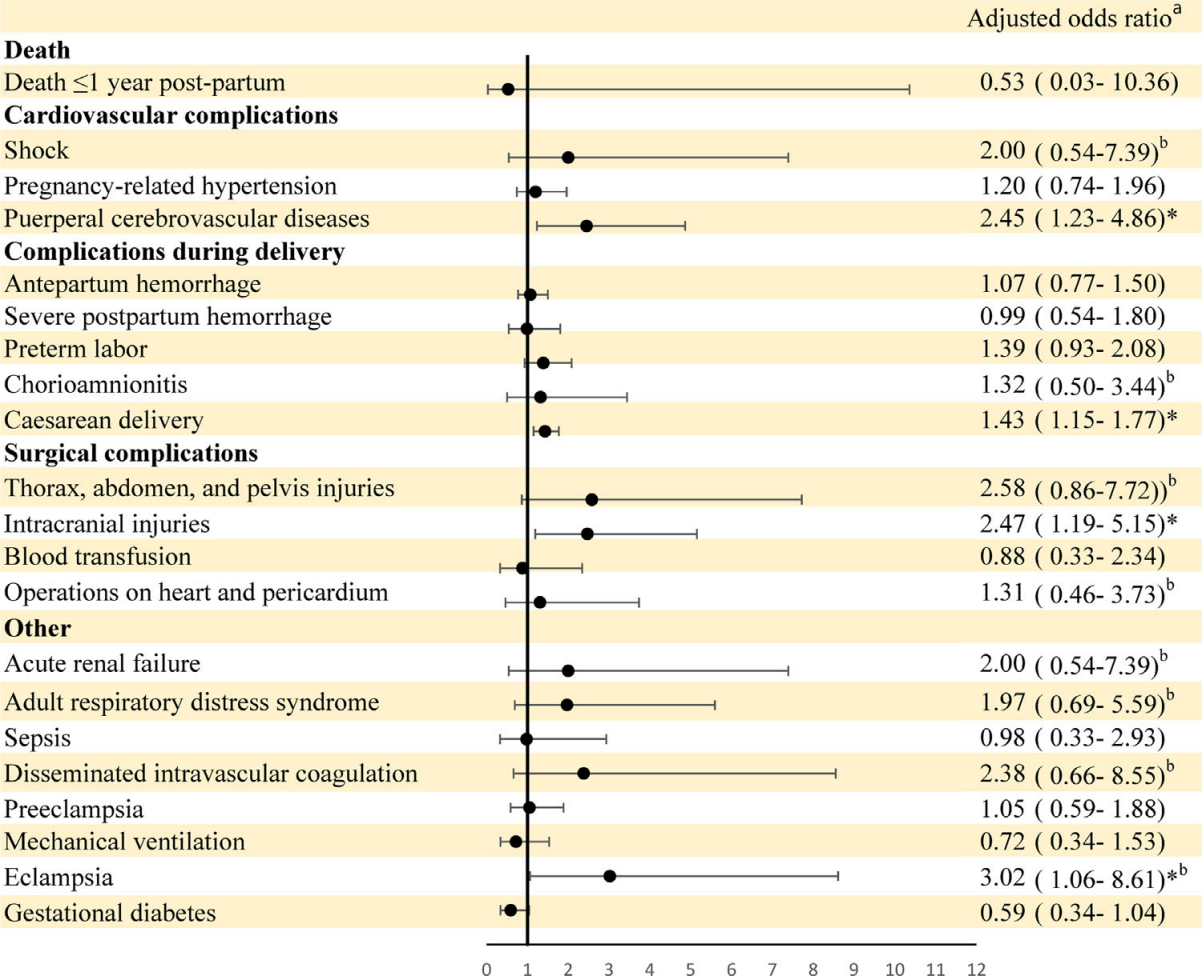
Comparison of pregnancy adverse outcomes between pregnant women with epilepsy with and without antiseizure medication use (nonuse = reference group) (*N* = 1400). ^a^Adjusted for age, infant sex, Charlson comorbidity index, urbanization, income, occupation, birth year, maternal nationality, maternal smoking, and maternal alcohol consumption. ^b^Bayesian logistic regression using data augmentation to avoid sparse data bias because of low numbers of events (*n* < 5). ^*^ *p* < .05.

To evaluate the impact of ASMs on pregnancy outcomes, the exposure and nonexposure groups were compared. The exposure group had higher adjusted odds ratios of eclampsia (OR: 3.02, 95% CI: 1.06−8.61), maternal intracranial injury (OR: 2.47, 95% CI: 1.19−5.15), puerperal cerebrovascular diseases (OR: 2.45, 95% CI: 1.23−4.86), and cesarean delivery (OR: 1.43, 95% CI: 1.15−1.77) (Figure 1).

### 3.2 Adverse fetal‐neonatal outcomes

Among the fetal‐neonatal adverse outcomes, small for gestational age was the most prevalent, while CNS malformation was the least prevalent. Compared with the control group, women with epilepsy had higher rates of low birth weight (< 2500 g), preterm labor, unexplained stillbirth, and CNS malformation. After adjusting for age, infant sex, maternal CCI, urbanization, income, occupation, birth year, maternal nationality, maternal smoking, and maternal alcohol consumption, the offspring of epileptic mothers were at higher risk of poor health outcomes. Specifically, the adjusted odds ratios for unexplained stillbirth in the exposure and nonexposure groups were 2.51 (95% CI: 1.41−4.48) and 2.37 (95% CI: 1.37−4.09), respectively. For CNS malformation, the ratios were 3.57 (95% CI: 1.86−6.86) and 2.25 (95% CI: 1.05−4.82), respectively. The odds ratios for low birth weight (< 2500 g) were 1.90 (95% CI: 1.48−2.44) and 1.97 (95% CI: 1.59−2.45), those for other/unspecified abnormalities were 1.58 (95% CI: 1.02−2.45) and 1.59 (95% CI: 1.06−2.37), and those for prematurity were 1.38 (95% CI: 1.06−1.81) and 1.56 (95% CI: 1.24−1.97) compared to the controls, respectively (Table 3).

**TABLE 3:** Fetal‐neonatal adverse outcomes among women without epilepsy (control), with epilepsy and taking (exposure) or not taking (nonexposure) antiseizure medications.

	No. of events (%)						Crude odds ratio (Control as reference group)				Adjusted odds ratio^a^ (Control as reference group)			
	Control (*n* = 2,004,479)		Nonexposure (*n* = 771)		Exposure (*n* = 629)		Nonexposure		Exposure		Nonexposure		Exposure	
Fetus outcome														
Stillbirth	15,293	(0.76)	14	(1.82)	13	(2.07)	2.44	(1.44–4.13)*	2.72	(1.58–4.68)*	2.31	(1.36–3.91)*	2.47	(1.42–4.29)*
Explained stillbirth	1556	(0.08)	1	(0.13)	1	(0.16)	1.68	(0.24–11.95)	2.06	(0.29–14.65)	1.67	(0.24–11.93)	1.97	(0.28–14.09)
							1.90	(0.65–5.50) ^b^	2.02	(0.68–6.02) ^b^	1.88	(0.65–5.44) ^b^	1.98	(0.67–5.86) ^b^
Unexplained stillbirth	13,737	(0.69)	13	(1.69)	12	(1.91)	2.52	(1.46–4.34)*	2.78	(1.57–4.91)*	2.37	(1.37–4.09)*	2.51	(1.41–4.48)*
Low birth weight (<2500 g)	131,300	(6.55)	104	(13.49)	86	(13.67)	2.22	(1.79–2.77)*	2.20	(1.72–2.82)*	1.97	(1.59–2.45)*	1.90	(1.48–2.44)*
Large for gestational age	197,043	(9.83)	76	(9.86)	50	(7.95)	1.01	(0.79–1.29)	0.80	(0.59–1.08)	1.04	(0.81–1.33)	0.80	(0.59–1.09)
Small for gestational age	196,540	(9.81)	99	(12.84)	97	(15.42)	1.34	(1.08–1.67)*	1.65	(1.32–2.06)*	1.24	(0.99–1.54)	1.51	(1.21–1.89)*
Decreased fetal movements	47,125	(2.35)	20	(2.59)	14	(2.23)	1.17	(0.75–1.81)	0.93	(0.53–1.64)	0.86	(0.55–1.35)	0.83	(0.47–1.46)
Fetal abnormalities, any	112,091	(5.59)	66	(8.56)	49	(7.79)	1.56	(1.21–2.03)*	1.43	(1.06–1.92)*	1.37	(1.05–1.78)*	1.33	(0.99–1.79)
Central nervous system malformations	8829	(0.44)	7	(0.91)	9	(1.43)	2.01	(0.94–4.30)	3.38	(1.76–6.49)*	2.25	(1.05–4.82)*	3.57	(1.86–6.86)*
Chromosomal abnormalities	9194	(0.46)	5	(0.65)	4	(0.64)	1.41	(0.57–3.49)	1.39	(0.53–3.66)	1.49	(0.60–3.70)	1.44	(0.54–3.84)
Unspecified abnormalities	42,777	(2.13)	29	(3.76)	22	(3.50)	1.73	(1.16–2.58)*	1.67	(1.08–2.59)*	1.59	(1.06–2.37)*	1.58	(1.02–2.45)*
							1.57	(0.78–3.19) ^b^	1.58	(0.74–3.36) ^b^	1.61	(0.79–3.27) ^b^	1.59	(0.75–3.40) ^b^
**Perinatal outcome**														
Preterm (<37 weeks)	150,978	(7.53)	95	(12.32)	74	(11.76)	1.70	(1.35–2.15)*	1.59	(1.22–2.06)*	1.56	(1.24–1.97)*	1.38	(1.06–1.81)*
Apgar score 1 min (< 7)	38,182	(1.90)	28	(3.63)	20	(3.18)	1.93	(1.31–2.86)*	1.69	(1.06–2.69)*	1.82	(1.23–2.68)*	1.51	(0.95–2.42)
Apgar score 5 min (< 7)	7828	(0.39)	9	(1.17)	3	(0.48)	2.95	(1.51–5.79)*	1.22	(0.38–3.86)	2.79	(1.43–5.45)*	1.08	(0.34–3.39)
							2.80	(1.52–5.14)* ^b^	1.51	(0.67–3.39) ^b^	2.63	(1.43–4.81)* ^b^	1.37	(0.61–3.05) ^b^
Fetal distress	98,121	(4.90)	31	(4.02)	26	(4.13)	0.80	(0.56–1.16)	0.82	(0.55–1.22)	0.79	(0.55–1.13)	0.79	(0.53–1.18)

^a^ Adjusted for age, infant sex, Charlson comorbidity index, urbanization, income, occupation, birth year, maternal nationality, maternal smoking, maternal alcohol consumption.

^b^ Bayesian logistic regression using data augmentation to avoid small sample bias because of the low occurrence of outcomes (number < 5).

*p < .05.

Relative to the controls, women with epilepsy exposed to ASMs had a higher risk of delivering infants classified as small for gestational age, with an adjusted odds ratio of 1.51 (95% CI: 1.21−1.89). Offspring of epileptic mothers not on ASMs had higher risks of lower Apgar scores at both 1 min (OR: 1.82, 95% CI: 1.23−2.68) and 5 min (OR: 2.63, 95% CI: 1.43−4.81), and a higher risk of fetal abnormalities (OR: 1.37, 95% CI: 1.05−1.78). Notably, no differences were seen in explained stillbirths or chromosome abnormalities between the epilepsy and control groups.

**FIGURE 2:**
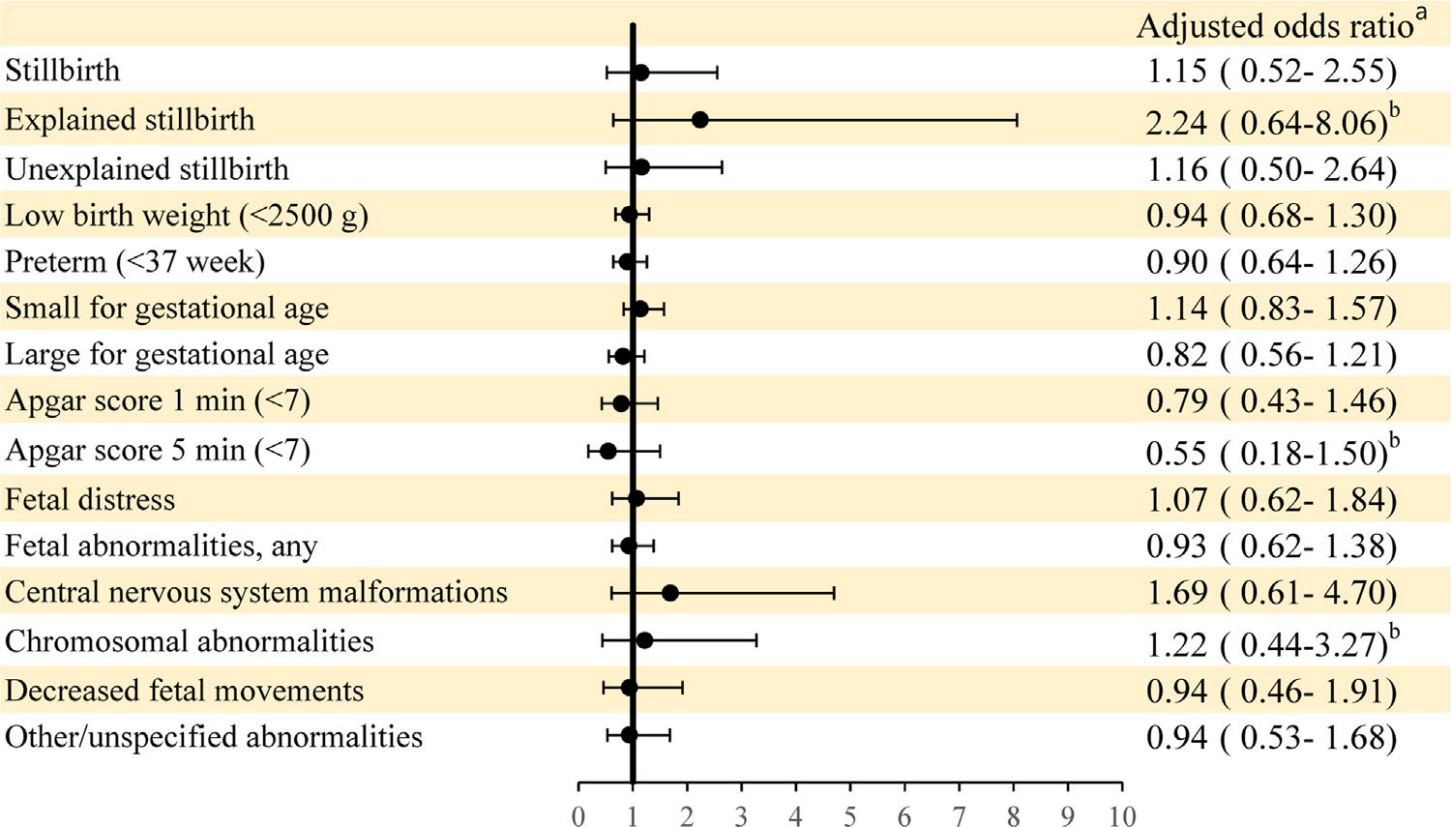
Comparison of fetal‐neonatal adverse outcomes between pregnant women with epilepsy with and without antiseizure medication use (nonuse = reference group) (*N* = 1400). ^a^Adjusted for age, infant sex, Charlson comorbidity index, urbanization, income, occupation, birth year, maternal nationality, maternal smoking, and maternal alcohol consumption. ^b^Bayesian logistic regression using data augmentation to avoid sparse data bias because of low numbers of events (*n* < 5).

Regarding the impact of ASMs on fetal and neonatal outcomes, no significant differences were observed between the exposure and nonexposure groups (Figure 2).

## 4 DISCUSSION

Over 90% of women with epilepsy have a straightforward delivery (Borthen et al., 2009). However, some face specific pregnancy‐related challenges, including preterm labor, gestational hypertension, preeclampsia, and antepartum hemorrhage. This study found that women with epilepsy, irrespective of whether they used ASMs, have an increased risk of complications during pregnancy and adverse outcomes for both the fetus and neonate. Some of these findings align with previous reports, while others are novel.

The risk of maternal death during pregnancy is generally low for women with epilepsy, at about 0.8 deaths per 1000 maternities (Macdonald et al., 2015). However, previous studies (Christensen et al., 2018; Edey et al., 2014; Macdonald et al., 2015; Yucel et al., 2021) revealed a 5‐ to 10‐fold increased risk of maternal death during pregnancy for women with epilepsy compared to the normal population. Maternal deaths associated with epilepsy accounted for 2.5%−5.3% of all maternal deaths (Yucel et al., 2021). Most cases were of sudden and unexpected death (SUDEP), which accounted for 79% of all deaths in one study (Edey et al., 2014). However, no consistent risk factors for SUDEP during pregnancy have been identified (Tanaka et al., 2021).

We found that women with epilepsy, irrespective of their use of ASMs, were at elevated risk of puerperal cerebrovascular diseases, adult respiratory distress syndrome, sepsis, pregnancy‐induced hypertension, preeclampsia, cesarean delivery, and preterm labor. Women with epilepsy and ASMs had a higher risk of puerperal cerebrovascular diseases, eclampsia, intracranial injuries, and cesarean delivery than those without ASMs.

Among our findings, the most noteworthy was that puerperal cerebrovascular complications had a high odds ratio, which had not been reported previously. Puerperal cerebrovascular diseases were identified in 0.07% of the women without epilepsy during pregnancy, similar to the rates in previous reports (0.008%–0.08%; Elgendy et al., 2020; Yger et al., 2021) and higher than that of women of reproductive age who are not pregnant (about 0.01%; Petitti et al., 1997). The presence of puerperal cerebrovascular disease in the current study was determined based on ICD‐9 codes for cerebrovascular diseases, including subarachnoid hemorrhage, intracerebral hemorrhage, occlusion of precerebral arteries and cerebral arteries, transient cerebral ischemia, venous complications in pregnancy and the puerperium, cerebrovascular disorders in the puerperium, peripheral vascular complications not classified elsewhere, and vascular complications of medical care not classified elsewhere (Table S2). Pregnancy is a risk factor for cerebrovascular diseases, which are predominantly associated with pregnancy‐induced complications, including preeclampsia, gestational hypertension, postpartum hemorrhage, postpartum infections, and fluid and electrolyte imbalances. Preexisting comorbidities, such as hypertension, cardiac conditions, thrombophilia, lupus, diabetes, migraines, alcohol consumption, and smoking, further contribute to this risk (James et al., 2005). In addition, individuals with epilepsy have a higher risk of cerebrovascular and cardiovascular diseases compared to the general population (Brodie et al., 2013). Preexisting cerebrovascular anomalies, such as AVMs, cerebral aneurysms, and venous irregularities, might worsen during pregnancy, trigger epilepsy onset, or amplify pregnancy‐associated risks. Furthermore, enzyme‐inducing ASMs (e.g., phenytoin and carbamazepine) and enzyme inhibitors (e.g., valproic acid) might amplify vascular risk by increasing the serum cholesterol level (Mintzer et al., 2009) or intima‐media thickness (Chuang et al., 2012). Anticonvulsants may interfere with coagulopathic factors; valproic acid is associated with thrombocytopenia (Buoli et al., 2018), causing a tendency toward bleeding.

Among fetal and neonatal complications, we found that the incidence of unexplained stillbirths was higher among fetuses from women with epilepsy, regardless of ASM exposure. Neonates born to mothers with epilepsy had a higher risk of low birth weight and preterm labor, consistent with previous reports (Viale et al., 2015).

We revealed a subtle trend toward teratogenic effects related to ASMs in cases with CNS malformations. However, the overall impact on comprehensive fetal anomalies was not significant. In our study population, the high‐risk teratogenic ASMs valproate (20.9%), topiramate (10.0%), and phenobarbital (4.1%) accounted for 35% of all observed cases. In comparison with the results from the UK Epilepsy and Pregnancy Register, which reported that a mean dose of 906.9 mg/day was associated with an elevated risk of major congenital malformations (MCMs), the daily dose in this study was lower. However, when valproate doses were within the range of 600−1000 mg/day, the MCM rate was approximately 6.1% (Campbell et al., 2014). Therefore, it is difficult to attribute these findings to the use of less teratogenic ASMs or lower doses alone. These results may be explained by the methodological limitations of our study. Our analysis was based exclusively on neonatal birth records, which are predominantly informed by physical examinations and antepartum sonographic evaluations. It is imperative to note that certain congenital heart diseases, laryngomalacia, spina bifida occulta, and chromosomal anomalies are typically diagnosed in outpatient departments and may not have been included in our assessment if not made antepartum. This suggests that our study underestimated the prevalence of certain congenital malformations. Our results align with a population‐based cohort study published in the Journal of JAMA Neurology (Razaz et al., 2017), in which the teratogenic effects of ASMs were not statistically significant.

Congenital malformation seems to be more associated with the presence of epilepsy rather than the administration of ASMs. We found that the incidence of fetal anomalies and congenital central nervous system malformations was higher in the offspring of women with epilepsy, irrespective of their exposure to ASMs, compared to the controls. It is important to note that this finding may not necessarily reflect a direct causal relationship. For instance, women with epilepsy may have a higher risk of developing preeclampsia, a condition that has previously been associated with microcephaly, hypospadias (Nelson et al., 2015), and noncritical congenital heart defects (Auger et al., 2015) in newborns.

This study, using a nationwide database, sought to provide insight into the potential risks associated with epilepsy and ASM use during pregnancy, and to improve maternal and neonatal care for women with epilepsy. It benefitted from an in‐depth analysis of various pregnancy and fetal‐neonatal outcomes related to epilepsy and ASM exposure during the first trimester of pregnancy, with adjustment for maternal socioeconomic status and comorbidity.

It is also necessary to acknowledge several limitations of this study. Concerning fetal and neonatal adverse outcomes, we relied solely on congenital malformation data from birth records. This approach may have led to an underestimation of malformation incidence since certain malformations can be identified only with brain magnetic resonance imaging. Moreover, we did not adjust for some potentially relevant covariates, such as maternal characteristics (parity, body weight, height, infection, the use of psychotropic or pain medications during pregnancy, and assisted pregnancy), pregnancy complications (gestational diabetes mellitus, pregnancy‐induced hypertension, preeclampsia, and eclampsia), and the occurrence of seizures during pregnancy. Notably, the presence of preeclampsia and seizures during pregnancy could play a vital role in the associations of epilepsy with adverse fetal and neonatal outcomes. Finally, we compared adverse outcomes between epilepsy patients with and without ASMs. We observed high rates of intracranial injury, puerperal cerebrovascular disease, and eclampsia in the ASM exposure group. However, it is important to note that the severity of epilepsy and seizure frequency during pregnancy may differ between these two groups. Further studies are needed to confirm whether the differences documented herein are attributable to ASM exposure or the frequency of seizures during pregnancy.

## 5 CONCLUSIONS

Compared to ASMs, epilepsy may have a stronger association with adverse maternal and neonatal outcomes. The potential for puerperal cerebrovascular complications in epileptic women might be underestimated. A vigilant and proactive approach is imperative to optimize both pregnancy and neonatal outcomes.

## AUTHOR CONTRIBUTIONS

IJ Chou, MJ Chiou, and CF Kuo designed the research concepts. MJ Chiou constructed the linkage system of all databases. IJ Chou, CF Kuo, CY Kuo, and MJ Chiou developed the research theory and prepared the grant application. CF Kuo and MJ Chiou performed all the statistical analyses and prepared figures and tables. LC See recommended and verified the analytical methods. IJ Chou, CY Kuo, CF Kuo, PC Hung, KL Lin, HS Wang, and JJ Lin provided clinical input and helped to interpret the data from the medical point of view. IJ Chou and CY Kuo wrote the first draft of the manuscript, and all authors gave their comments on the manuscript and critically revised the intellectual contents. IJ Chou had full access to all the data in the study and took responsibility for the integrity of the data and the accuracy of the data analysis. The corresponding author (IJ Chou) attests that all listed authors meet authorship criteria and that no others meeting the criteria have been omitted.

## FUNDING

This work was funded by the National Science Council of Taiwan (project 104‐2314‐B‐182A‐047) and Chang Gung Memorial Hospital (project CORPG3G0411, CORPG3G0461). The sponsors of the study, the Chang Gung Memorial Hospital, and the National Science Council had no role in the design and conduct of the study; collection, management, analysis, and interpretation of the data; preparation, review, or approval of the manuscript; and decision to submit the manuscript for publication. The interpretation and conclusions contained herein do not represent those of the National Health Insurance Administration, Ministry of Health and Welfare, Taiwan.

## CONFLICT OF INTEREST STATEMENT

The authors have no conflict of interest to declare.

## ETHICS STATEMENT

This study was approved by the Institutional Review Board of Chang Gung Medical Foundation (approval number 201701015B0) and the National Health Insurance Administration, Department of Health and Welfare (the custodian of the NHIRD). All data in this study were anonymized by the Ministry of Health and Welfare. Therefore, the need for patient consent was waived.

### PEER REVIEW

The peer review history for this article is available at https://publons.com/publon/10.1002/brb3.3287.

## Supporting information

Figure S1 Information

Figure S2 Information

Figure S3 Information

Table S1 Information

Table S2 Information

Table S3 Information

## Data Availability

The source data of this study is the NHI database, which has limited access to investigators having ethical and administrative approval. The lead author (I.J. Chou) affirms that the manuscript is an honest, accurate, and transparent account of the study being reported; no important aspects of the study have been omitted. Any discrepancies from the study as planned have been omitted and explained. The code is available on request from the corresponding author.
