# Supplementary material for: The impact of epilepsy and antiseizure medications on pregnancy and neonatal outcomes: A nationwide cohort study
Source: Brain Behav. 2023 Oct 14;13(12):e3287. doi: 10.1002/brb3.3287 (PMC10726760; doi:10.1002/brb3.3287)

Figure 1. Study Design and Cohort Flow Chart

Exposure = pregnant women with epilepsy and taking anti-seizure medications

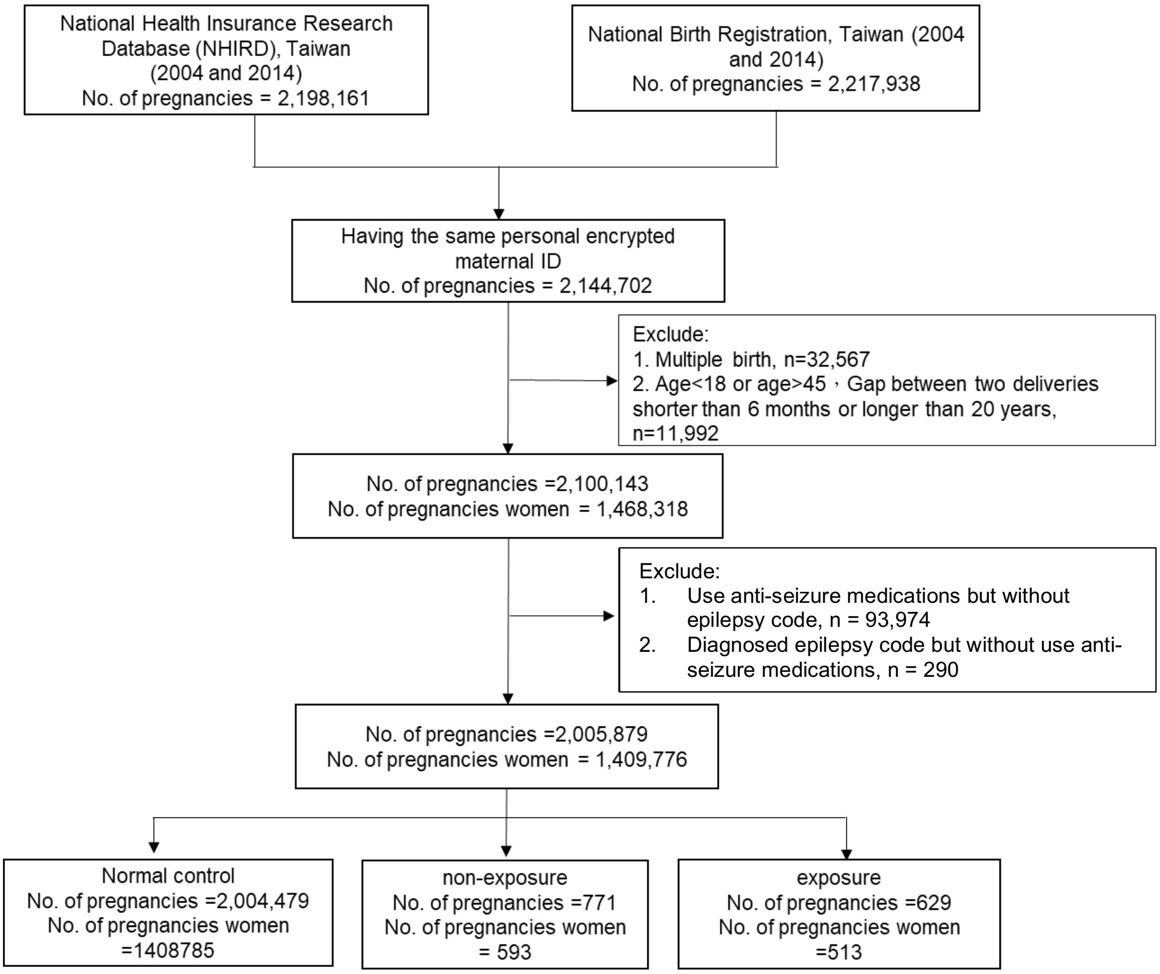

Supplement: Supplementary file 1 — Figure S1 Information [file BRB3-13-e3287-s003.pdf]
