# Supplementary figures and images for: The impact of epilepsy and antiseizure medications on pregnancy and neonatal outcomes: A nationwide cohort study
Source: Brain Behav. 2023 Oct 14;13(12):e3287. doi: 10.1002/brb3.3287 (PMC10726760; doi:10.1002/brb3.3287)

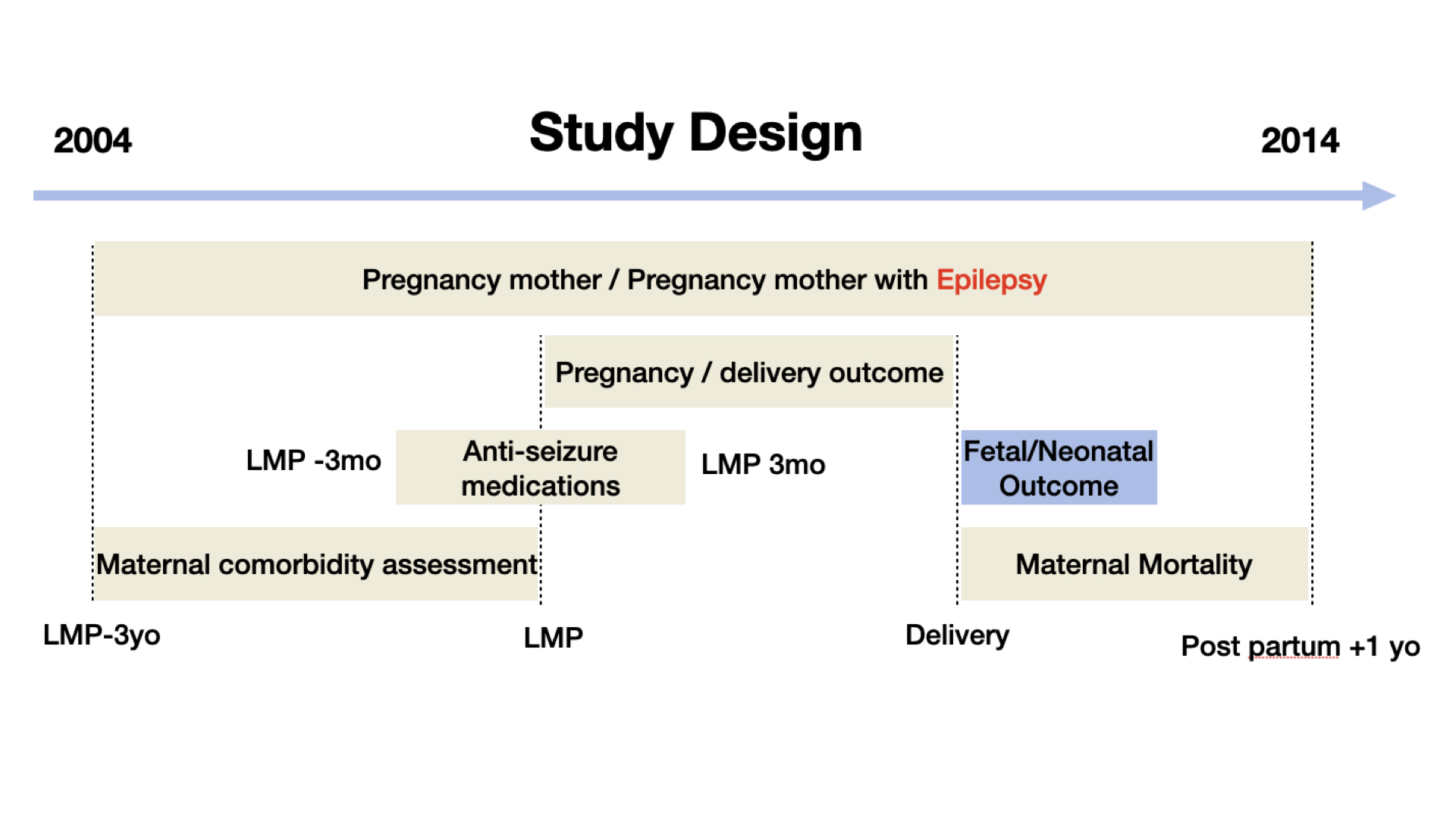

Supplement: Supplementary file 2 — Figure S2 Information [file BRB3-13-e3287-s004.jpeg]
