# Supplementary material for: The impact of epilepsy and antiseizure medications on pregnancy and neonatal outcomes: A nationwide cohort study
Source: Brain Behav. 2023 Oct 14;13(12):e3287. doi: 10.1002/brb3.3287 (PMC10726760; doi:10.1002/brb3.3287)
Supplement: Supplementary file 3 — Figure S3 Information [file BRB3-13-e3287-s006.docx]

Fig 3. Nomogram for birth weight in Taiwan summarised from National Birth Registry 2001-2012.


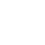

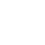

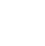

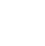

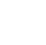

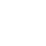

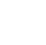

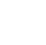

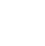

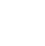

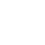

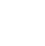

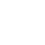

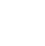

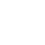

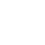

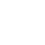

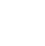

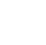

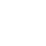

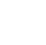

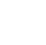

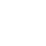

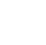

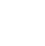

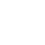

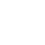

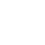

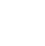

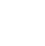

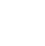

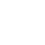

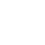

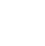

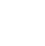

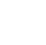

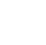

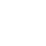

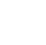

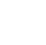

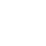

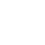

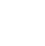

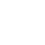

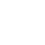

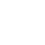

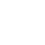

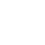

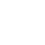

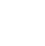

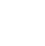

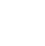

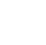

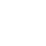

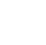

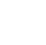

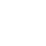

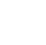

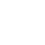

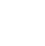

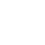

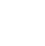

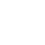

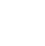

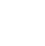

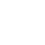

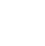

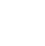

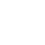

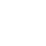

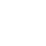

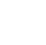

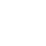

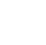

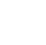

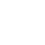

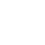

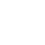

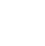

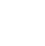

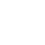

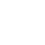


**3550 3650 3510 3600 3650**

**3751 3846 3939**

**3198 3166 3078 3180 3040 3100 3100**

**3400 3300 3420 32763341.53375**

**2680 2690 2680 2700 2650 2750**

**2440 2450 2450 2440 2484**

**2204 2208 2210 2200 2200**

**2938 2930 2920 2850 2950 2840 2900 2898**

**1840 1800 1800 1780 1750**

**1992 2000 2000 1995 1982**

**1404 1435 1445**

**1600 1626 1600 1600 1532**

**1907700 1100 1110 1100 1046 1184**

**1226.51260 1284 1250 1404 1344**

**850 985.5 960**

**732**

**850**

**580**

**670**

**760**

**890**

26 27 28 29 30 31 32 33 34 35 36 37 38 39 40 41 42

Gestational age (week)

10%

25%

50%

75%

90%

Birth weught (gm)
