# Supplementary material for: The impact of epilepsy and antiseizure medications on pregnancy and neonatal outcomes: A nationwide cohort study
Source: Brain Behav. 2023 Oct 14;13(12):e3287. doi: 10.1002/brb3.3287 (PMC10726760; doi:10.1002/brb3.3287)
Supplement: Supplementary file 4 — Table S1 Information [file BRB3-13-e3287-s001.docx]

Table 1. Anti-seizure medications (ASMs) codes

| **Classification** | **ATC Code** |
| --- | --- |
| **Sodium Channel Blockers** |  |
| Carbamazepine | N03AF01 |
| Phenytoin | N03AB02 |
| Oxcarbazepine | N03AF02 |
| Lamotrigine | N03AX09 |
| Zonisamide | N03AX15 |
| **GABA Receptor Agonists** |  |
| Clobazam | N05BA09 |
| Clonazepam | N03AE01 |
| Phenobarbital | N03AA02 |
| Primidone | N03AA03 |
| **GABA Reuptake Inhibitors** |  |
| Tiagabine | N03AG06 |
| **GABA Transaminase Inhibitors** |  |
| Vigabatrin | N03AG04 |
| **ASMs with Potential GABA Mechanism of Action** |  |
| Gabapentin | N03AX12 |
| Pregabalin | N03AX16 |
| Valproate | N03AG01 |
| **Glutamate Blockers** |  |
| Topiramate | N03AX11 |
| **ASMs with Other Mechanisms of Action** |  |
| Levetiracetam | N03AX14 |
| **Acetazolamide** | S01EC01 |
