# Supplementary material for: The impact of epilepsy and antiseizure medications on pregnancy and neonatal outcomes: A nationwide cohort study
Source: Brain Behav. 2023 Oct 14;13(12):e3287. doi: 10.1002/brb3.3287 (PMC10726760; doi:10.1002/brb3.3287)
Supplement: Supplementary file 5 — Table S2 Information [file BRB3-13-e3287-s002.docx]

Table 2. Codes used to identify fetal-neonatal and maternal outcomes

| **Disease definition** | |
| --- | --- |
| Deliver | DRG code: 0371A, 0371a, 0373B, 0373b, 0373A, 0373a, 0373C, 0373c  Icd-9：V27, 650, 651, 652  Icd-op-code：72, 73, 74 |
| **Cardiovascular** |  |
| Acute myocardial | 410.xx |
| Aneurysm | 441.xx |
| Cardiac arrest/ ventricular  fibrillation | 427.41, 427.42, 427.5 |
| Heart failure | 669.4x, 997.1 |
| Shock | 669.1x, 785.5x |
| Conversion of cardiac  rhythm | 99.6x |
| Pregnancy-related  hypertension | 642.3x 642.9x 642.4x 642.5x 642.6x 642.7x |
| Gestational hypertension | 642.3x 642.9x |
| Puerperal cerebrovascular  disorders | 430, 431, 432.x, 433.xx, 434.xx, 436, 437.x, 671.5x, 674.0x,  997.2, 999.2 |
| Thrombotic embolism | 415.1x, 673.0x, 673.2x, 673.3x, 673.8x, |
| **Complication during**  **delivery** |  |
| Amniotic fluid embolism | 673.1x |
| Antepartum hemorrhage | 641.1x 641.2x 641.3x 641.8x 641.9x |
| Postpartum hemorrhage due  to atony | 666.1x |
| Postpartum hemorrhage not  due to atony | 666.0x 666.2x 666.3x |
| Severe postpartum  hemorrhage | [666.0x-666.3x] plus either blood transfusion [99.0x] or  hysterectomy [68.3x-68.9] |
| Preterm labor | 644.2x |
| Premature rupture of  membranes | 658.1x |
| Chorioamnionitis | 658.4x |
| Cesarean delivery | Birth Registration |
| Induction of labor | 73.1 73.4 |

| **Surgical complications** |  |
| --- | --- |
| Severe anesthesis  complications | 668.0x, 668.1x, 668.2x |
| Thorax, abdomen, and  pelvis injuries | 860.xx-869.xx |
| Intracranial injuries | 800.xx, 801.xx, 803.xx, 804.xx, 851.xx, 854.xx |
| Blood transfusion | 99.0x |
| Hysterectomy | 68.3x-68.9 |
| Operations on heart and  pericardium | 35.xx, 36.xx, 37.xx, 39.xx |
| **Other** |  |
| Acute renal failure | 584.x, 669.3x |
| Adult respiratory distress  syndrome | 518.5, 518.81, 518.82, 518.84, 799.1 |
| Sepsis | 038.xx, 995.91, 995.92 |
| Disseminated intravascular  coagulation | 286.6, 286.9, 666.3x |
| Preeclampsia | 642.4x 642.5x 642.6x 642.7x |
| pulmonary edema | 428.1, 518.4 |
| Temporary tracheostomy | 31.1 |
| Ventilation | 93.90, 96.01-96.05, 96.7x |
| Seizures among  preeclamptic patients | 642.6x |
| Gestational diabetes | 648.8x |
| Low birth weight | Birth Registration, <2500 g |
| Preterm labor | Birth Registration, <37 week |
| Fetal distress | 656.3x, 659.7x |
| Fetal abnormalities, any | Birth Registration + inpatient claim data (icd9 (655.xx)) |
| Central nervous system  malformations | 655.0x |
| Chromosomal abnormalities | 655.1x |
| Hereditary disease in family  possible affecting fetus | 655.2 |
| Suspected damage due to  viral or other diseases in the mother | 655.3x 655.4x |
| Suspected damage due to | 655.5x 655.6x |

| drugs or radiation |  |
| --- | --- |
| Decreased fetal movements | 655.7x |
| Other/unspecified  abnormalities | 655.8x 655.9x |
| Stillbirth | Birth Registration |
