# Supplementary material for: The impact of epilepsy and antiseizure medications on pregnancy and neonatal outcomes: A nationwide cohort study
Source: Brain Behav. 2023 Oct 14;13(12):e3287. doi: 10.1002/brb3.3287 (PMC10726760; doi:10.1002/brb3.3287)
Supplement: Supplementary file 6 — Table S3 Information [file BRB3-13-e3287-s005.docx]

Supplementary Table 3.

The detailed information on anti-seizure medications in the exposure group.

| No of drugs | % |
| --- | --- |
| 1 | 63.8 |
| 2 | 23.7 |
| 3 | 12.5 |
| Drug name | % |
| Carbamazepine | 37.7 |
| Lamotrigine | 23.7 |
| Valproate | 20.9 |
| Clonazepam | 18.3 |
| Phenytoin | 16.2 |
| Oxcarbazepine | 12.0 |
| Topiramate | 10.0 |
| Phenobarbital | 4.1 |
| Gabapentin | 4.0 |
| Clobazam | 2.9 |
| Acetazolamide | 1.6 |
| Vigabatrin | 1.5 |
| Pregabalin | 0.2 |
| Tiagabine | 0.1 |
| Perampanel | 0.0 |
| Primidone | 0.0 |
| Zonisamide | 0.0 |
